# The Implementation of a New Inpatient Admission Sheet in the Department of Pediatrics at the Atbara Teaching Hospital: A Quality Improvement Project

**DOI:** 10.7759/cureus.90870

**Published:** 2025-08-24

**Authors:** Saifalden Mustafa Badralden Mustafa, Abubakr Muhammed, Algazali Abdelrahman Ahmed Jadalla, Abubaker Mahmood Mohamed Osman, Ibrahim Abdelhay Ibrahim Alboshary, Mohammed Abdelmagid Yousif Ahmed, Abduldafi Galal Mohammed El Siddig, Doaa Mahmoud Akasha Mohamed, Nuha Hussain Abdallah Ahmed, Mustafa Ahmed, Alkhir Ali Abadalkareem Alhassan, Salaheldin Ahmed, Wagar Ishag Yagoub Shaddad, Athiel Kamal Eldeen Fadul Osman, Sara Mohamedosman Mohamed Ahmed Hamid, Weaam Mohammed Awad Ahmed, Aiat Osama Elmabrouk Hassan, Nada Salaheldin Suliman Ali, Egbal Sahal Abdelmaged Ibrahim, Marwa Mohamed

**Affiliations:** 1 Pediatrics, Atbara Teaching Hospital, Atbara, SDN; 2 Surgery, University of Gezira, Madani, SDN; 3 Community Medicine, Atbara Teaching Hospital, Atbara, SDN; 4 Surgery, Atbara Teaching Hospital, Atbara, SDN; 5 Pediatrics, University of Gezira, Madani, SDN; 6 Pediatrics, CHI at Temple Street, Dublin, IRL; 7 Pediatrics, Ambulatory Healthcare Services, Abu Dhabi, ARE; 8 Pediatrics, Prince Charles Hospital, Merthyr Tydfil, GBR; 9 Pediatrics, Omdurman Islamic University, Khartoum, SDN; 10 Pediatrics, University of Dongola, Dongola, SDN; 11 Pediatrics, Hamad Medical Corporation, Doha, QAT; 12 Emergency Department, Burjeel Hospital, Abu Dhabi, ARE; 13 Pediatrics, Elrazi University, Khartoum, SDN

**Keywords:** care continuity, department of paediatrics, medical record documentation, patient safety, quality improvement projects

## Abstract

Background

Inadequate medical record documentation poses significant risks to patient safety and care continuity. This study aimed to analyze a quality improvement project that involved the implementation of a standardized inpatient admission sheet in the Department of Pediatrics at Atbara Teaching Hospital, Sudan. The project sought to address critical gaps in documentation completeness and accuracy.

Methods

We assessed a one-year quality improvement project (July 2024-July 2025) involving three audit cycles with two intervening interventions comprising staff training, standardized documentation protocols, checklist prompts, and enhanced supervision. Completeness rates for 25 admission parameters were evaluated across 153 pediatric admission records (Cycle 1: n=53; Cycle 2: n=50; Cycle 3: n=50). Statistical analysis was performed using chi-square (χ²) or Fisher’s exact tests with significance set at p<0.05.

Results

At baseline (Cycle 1), documentation deficiencies were profound, with 0% completion of data on gender, triage, family/past medical/social histories, and most physical examinations. Anthropometric measurements (e.g., height and mid-upper-arm circumference (MUAC) were documented in under 5%. Following the first intervention (Cycle 2), significant improvements occurred in gender documentation (0% to 34.0%; χ²=19.8, p<0.001), past medical history (0% to 68.0%; χ²=56.2, p<0.001), and cardiovascular examinations (7.5% to 68.0%; χ²=45.2, p<0.001). MUAC documentation increased to 20.0%. After the second intervention (Cycle 3), core demographic parameters, including name, age, gender, weight, and date, reached near-complete documentation (84.0% to 96.0%), and address and triage improved significantly. However, detailed clinical histories and system examinations regressed to 0% completion. Vital signs, allergy, and discharge status documentation remained absent across all cycles.

Conclusions

Implementation of standardized documentation tools combined with targeted training substantially enhanced key demographic and presenting complaint documentation in pediatric admissions. Nonetheless, sustaining comprehensive clinical history and physical examination recording remains challenging, necessitating ongoing reinforcement, simplification of documentation workflows, and potential integration of electronic solutions to ensure durable quality improvements.

## Introduction

Effective medical record keeping is an indispensable component of high-quality healthcare delivery. Comprehensive, clear, and contemporaneous documentation facilitates seamless communication among healthcare professionals, supports informed clinical decision-making, and ensures continuity of patient care. Abdelrahman and Abdelmageed underscore the pivotal importance of clarity, accuracy, and timeliness in medical records, as these attributes directly influence patient safety and overall health outcomes [1]. Conversely, incomplete or inaccurate records are associated with increased risk of medical errors and adverse events, underscoring the urgency of implementing continuous quality improvement measures in documentation practices.

Empirical research has identified significant challenges related to the completeness and standardization of medical records, particularly discharge summaries, across diverse healthcare environments. Bayisa et al. reported that multifaceted quality improvement interventions markedly improved the completeness of medical records in an Ethiopian referral hospital setting [2]. Similarly, audits conducted by Ge et al. and Eissa et al. revealed inconsistencies and deficiencies in documentation practices, while demonstrating the effectiveness of audit cycles in enhancing discharge summary quality [3-4]. Technological advancements, including the adoption of electronic medical record systems, coupled with compliance monitoring programs, have further contributed to improvements in documentation integrity [5-7]. More recently, Muhammed et al. have highlighted the benefits of structured documentation tools such as follow-up checklists and standardized discharge documentation in specialized hospital settings, which facilitate improved clinical communication and patient management [8,9].

In this context, the present study examines the implementation of a novel inpatient admission sheet within the Department of Pediatrics at Atbara Teaching Hospital. This initiative aims to address deficiencies in data capture and documentation quality, thereby aligning with broader objectives related to enhancing clinical information management, patient safety, and care coordination. The endeavor aligns with the overarching healthcare objective of achieving the Triple Aim, improving patient experience, population health, and reducing cost of care as articulated by Berwick et al. (3). Through systematic documentation improvement, healthcare institutions can significantly enhance clinical outcomes and operational efficiency.

## Materials and methods

This quality improvement project was conducted in the Department of Pediatrics at Atbara Teaching Hospital, Sudan, aiming to enhance inpatient admission documentation. The study spanned one year, from July 17, 2024, to July 17, 2025, and included a three-cycle quality improvement project with two intervening improvement phases. An observational approach was employed, focusing on continuous process improvement supported by active stakeholder engagement and feedback.

To support this initiative, the study team developed a new standardized inpatient admission sheet through a collaborative process involving pediatric specialists and key stakeholders. Historically, admission documentation had been handwritten on plain (blank) paper, resulting in inconsistent and incomplete records. The newly designed sheet (see figures in Appendices) introduced structured fields aligned with local documentation standards and clinical practice needs. It aimed to promote comprehensive data capture and improve the consistency and legibility of clinical entries across the department.

First cycle (pre-intervention, n=53): July 17 - October 31, 2024

The first cycle served as the baseline audit of existing admission documentation practices. Records of 53 admissions were reviewed using the new assessment framework. No structured admission form was in use at that time; documentation was typically handwritten.

First Intervention (November 1 - December 31, 2024)

A two-month targeted intervention phase followed, focusing on staff training, standardizing documentation protocols, and raising awareness about the importance of comprehensive data capture.

Second cycle (post-first Intervention, n=50): January 1 - March 31, 2025

The second cycle reviewed 50 records following the first intervention to assess improvements in documentation practices.

Second Intervention (April 1 - May 31, 2025)

To consolidate these improvements, a second intervention was implemented. This included reinforced training, refinement of the admission sheet documentation tools, introduction of checklist prompts, and enhanced supervisory oversight to drive compliance and completeness across all key clinical fields.

Third cycle (post-second intervention, n=50): June 1 - July 17, 2025

The third cycle reviewed 50 records to assess the impact of the second intervention.

Data analysis

Admission forms from all three cycles were reviewed for completeness and accuracy using predefined criteria. Completion rates for each parameter were calculated to evaluate improvements following interventions. Staff feedback was also analyzed qualitatively to identify challenges and inform further refinements. This combined approach guided ongoing enhancements to documentation practices.

Evaluation

The evaluation phase focused on assessing the impact of the interventions. It included reviewing feedback, identifying strengths and weaknesses in the new form's implementation, and gathering suggestions for future enhancements.

Ethical considerations

This study was reviewed by the Ethical Committee of Atbara Teaching Hospital and was deemed exempt from formal ethical approval, as it involved retrospective analysis of de-identified pediatric admission records without handling patient-identifiable data

## Results

In the first audit cycle (pre-intervention, n=53), the documentation of key patient admission parameters revealed significant deficiencies across nearly all assessed fields (Table 1). Basic demographic data such as gender and triage were completely absent (0/53, 0.0%), while address was documented in only 2/53 (3.8%) of cases. Although name and age were relatively well recorded (45/53, 84.9% each), important clinical history domains-including family history, past medical history, and social history-were entirely missing from all admission sheets (0/53, 0.0%). Similarly, documentation of physical examination findings for cardiovascular, respiratory, and gastrointestinal systems was minimal (each approximately 4/53, 7.5%). Basic anthropometric measurements like mid-upper-arm circumference (MUAC) and height were seldom recorded (2/53, 3.8%, and 0/53, 0.0%, respectively), whereas weight was documented in 41/53 (77.4%) of forms. These gaps highlighted the absence of a standardized admission documentation process and underscored an urgent need for intervention.

**Table 1:** Documentation compliance across three cycles CNS: central nervous system; CVS: cardiovascular; GIT: gastrointestinal tract; GU: genitourinary; IDP: internally displaced person; MUAC: mid-upper-arm circumference

Parameter	First cycle (n=53), n (%)	Second cycle (n=50), n (%)	Third cycle (n=50), n (%)	C1 vs. C2 statistic (p-value)	C2 vs. C3 statistic (p-value)	Notes
Triage	0 (0.0%)	1 (2.0%)	7 (14.0%)	Fisher's exact; p=1.000	Fisher's exact; p=0.055	Moderate improvement
Name	45 (84.9%)	47 (94.0%)	48 (96.0%)	Fisher's exact; p=0.617	Fisher's exact; p=1.000	Consistently high
Age	45 (84.9%)	47 (94.0%)	48 (96.0%)	Fisher's exact; p=0.617	Fisher's exact; p=1.000	Consistently high
Address	2 (3.8%)	1 (2.0%)	19 (38.0%)	Fisher's exact; p=1.000	χ²=21.0; p<0.001	Significant improvement
Gender	0 (0.0%)	17 (34.0%)	42 (84.0%)	χ²=19.8; p<0.001	χ²=29.6; p<0.001	Major improvement
Date	45 (84.9%)	41 (82.0%)	48 (96.0%)	χ²=1.9; p=0.168	χ²=7.1; p=0.008	Recovered/improved
Guardian relationship	0 (0.0%)	7 (14.0%)	0 (0.0%)	Fisher's exact; p=0.012	Fisher's exact; p=0.012	Regression
IDP status	0 (0.0%)	6 (12.0%)	0 (0.0%)	Fisher's exact; p=0.027	Fisher's exact; p=0.027	Regression
MUAC	2 (3.8%)	10 (20.0%)	0 (0.0%)	Fisher's exact; p=0.028	χ²=11.1; p=0.001	Regression
Weight	41 (77.4%)	39 (78.0%)	48 (96.0%)	χ²=0.3; p=0.581	χ²=10.9; p=0.001	Fully recorded
Height	0 (0.0%)	2 (4.0%)	0 (0.0%)	Fisher's exact; p=0.494	Fisher's exact; p=0.494	Regression
Chief complaint	45 (84.9%)	47 (94.0%)	48 (96.0%)	Fisher's exact; p=0.617	Fisher's exact; p=1.000	Consistently high
History of presenting illness	42 (79.2%)	45 (90.0%)	48 (96.0%)	χ²=1.1; p=0.294	χ²=3.3; p=0.071	Fully recorded
Family history	0 (0.0%)	24 (48.0%)	0 (0.0%)	χ²=29.5; p<0.001	χ²=29.5; p<0.001	Regression
Past medical history	0 (0.0%)	34 (68.0%)	0 (0.0%)	χ²=56.2; p<0.001	χ²=56.2; p<0.001	Regression
Social history	0 (0.0%)	21 (42.0%)	0 (0.0%)	χ²=25.6; p<0.001	χ²=25.6; p<0.001	Regression
CVS examination	4 (7.5%)	34 (68.0%)	0 (0.0%)	χ²=45.2; p<0.001	χ²=56.2; p<0.001	Regression
Respiratory examination	4 (7.5%)	35 (70.0%)	0 (0.0%)	χ²=47.1; p<0.001	χ²=58.3; p<0.001	Regression
GIT examination	4 (7.5%)	34 (68.0%)	0 (0.0%)	χ²=45.2; p<0.001	χ²=56.2; p<0.001	Regression
GU examination	0 (0.0%)	6 (12.0%)	0 (0.0%)	Fisher's exact; p=0.027	Fisher's exact; p=0.027	Regression
CNS examination	0 (0.0%)	5 (10.0%)	0 (0.0%)	Fisher's exact; p=0.056	Fisher's exact; p=0.056	Regression
MS examination	0 (0.0%)	4 (8.0%)	0 (0.0%)	Fisher's exact; p=0.117	Fisher's exact; p=0.117	Regression
Diagnosis	45 (84.9%)	47 (94.0%)	48 (96.0%)	Fisher's exact; p=0.617	Fisher's exact; p=1.000	Consistently high
Management plan	45 (84.9%)	47 (94.0%)	48 (96.0%)	Fisher's exact; p=0.617	Fisher's exact; p=1.000	Consistently high
Vitals	0 (0.0%)	0 (0.0%)	0 (0.0%)	-	-	Critical gap
Allergies	0 (0.0%)	0 (0.0%)	0 (0.0%)	-	-	Critical gap
Discharge status	0 (0.0%)	0 (0.0%)	0 (0.0%)	-	-	Critical gap

Following the implementation of the first intervention, which comprised targeted staff training and the introduction of a standardized inpatient admission sheet, documentation completeness improved markedly in the second audit cycle (post-first intervention, n=50). Documentation of patient demographic fields, such as gender, increased substantially to 17/50 (34.0%; χ²=19.8, p<0.001), while triage documentation showed a modest rise to 1/50 (2.0%; Fisher’s exact p=1.000). Clinical history documentation also improved considerably: past medical history was recorded in 34/50 (68.0%; χ²=56.2, p<0.001), family history in 24/50 (48.0%; χ²=29.5, p<0.001), and social history in 21/50 (42.0%; χ²=25.6, p<0.001) of cases. Documentation of physical examination parameters related to cardiovascular, respiratory, and gastrointestinal systems rose sharply to approximately 68-70%. Anthropometric data such as MUAC increased to 10/50 (20.0%; Fisher’s exact p=0.028). Core identifiers like name, age, and date remained consistently high (above 80%). These improvements reflected enhanced awareness and adoption of standardized recording practices. A second intervention was subsequently implemented, aiming to reinforce these gains through refresher training, refinement of documentation tools, introduction of checklist prompts, and closer supervisory oversight.

In the third audit cycle (post-second intervention, n=50), documentation of core patient identifiers reached near-complete levels: name, age, weight, and date all achieved 96.0% (48/50) completeness; gender documentation increased substantially to 42/50 (84.0%; χ²=29.6, p<0.001); address documentation improved significantly to 19/50 (38.0%; χ²=21.0, p<0.001); and triage rose to 7/50 (14.0%; Fisher’s exact p=0.055). However, a notable regression occurred in the documentation of detailed clinical history and physical examination parameters. Fields such as guardian relationships, IDP status, MUAC, past medical history, family history, social history, and all system examination records dropped back to 0/50 (0.0%) completion despite prior improvements (all χ²/Fisher’s exact tests, p<0.001). Meanwhile, documentation of key presenting complaints remained consistently strong, with the chief complaint and history of presenting complaint reaching 48/50 (96.0%). This pattern suggests challenges in sustaining comprehensive clinical documentation over time despite demonstrable improvements in basic demographic and complaint-related data.

Stakeholder feedback indicated high acceptance of the new admission sheet and training efforts, although some users requested further simplification of clinical fields and additional support mechanisms to maintain consistent use. In response, ongoing supervision, repeated audit cycles, and iterative tool refinement have been planned to ensure sustained quality and completeness of patient admission records. Table 2 presents the mean compliance change across the three documentation cycles.

**Table 2:** Mean compliance improvements across cycles

Comparison	Mean compliance (Cycle A)	Mean compliance (Cycle B)	Mean difference (%)	Direction of change
First vs. second cycle	34.7%	41.2%	+6.5%	Moderate overall improvement
Second vs. third cycle	41.2%	43.6%	+2.4%	Slight improvement
First vs. third cycle	34.7%	43.6%	+8.9%	Notable overall improvement

## Discussion

This quality improvement project aimed to enhance the completeness and accuracy of inpatient admission documentation in the Department of Pediatrics at Atbara Teaching Hospital through the implementation of a standardized admission sheet, targeted staff training, and ongoing audit cycles. The baseline assessment revealed critical gaps in documentation (Table 1), particularly in demographic details such as gender and triage categorization (0/53, 0.0% for each), and in key clinical information including family history, past medical history, and physical examination findings (e.g., family history: 0/53, 0.0%; cardiovascular examination: 4/53, 7.5%). These deficiencies are consistent with prior reports highlighting suboptimal medical record completeness in similar resource-constrained settings [1-4].

The first intervention, focusing on staff education and the introduction of a standardized form, resulted in substantial and statistically significant improvements across many documentation domains (Table 1). Notably, records of gender increased from 0/53 (0.0%) to 17/50 (34.0%) (χ²=19.8, p<0.001), past medical history from 0/53 to 34/50 (68.0%) (χ²=56.2, p<0.001), family history from 0/53 to 24/50 (48.0%) (χ²=29.5, p<0.001), and documentation of the cardiovascular system examination from 4/53 (7.5%) to 34/50 (68.0%) (χ²=45.2, p<0.001). These findings, incorporating both absolute numbers and percentages alongside relevant statistical test values and p-values, reflect statistically robust improvements (p<0.05 throughout) and align with existing literature demonstrating that targeted educational interventions combined with standardized tools can yield measurable gains in medical record quality [5-8]. A similar upward trend was evident for anthropometric data, with MUAC documentation improving from 2/53 (3.8%) to 10/50 (20.0%) (Fisher’s exact test; p=0.028), highlighting the value of incorporating specific clinical prompts within admission documentation forms.

In the third audit cycle, following an additional intervention-including refresher training, documentation tool refinement, and enhanced supervisory support-improvements in demographic and presenting complaint documentation were sustained or further advanced. For instance, gender documentation reached 42/50 (84.0%; χ²=29.6, p<0.001), address improved to 19/50 (38.0%; χ²=21.0, p<0.001), and name, age, date, and weight each achieved 48/50 (96.0%) completeness. However, a notable and statistically significant regression occurred in the recording of detailed clinical histories and system-based examinations, with family, social, and past medical histories, as well as all system examinations, dropping back to 0/50 (0.0%) despite earlier gains (χ² and Fisher’s exact tests, p<0.001; Table 1). This pattern may reflect documentation fatigue, workflow disruptions, or insufficient reinforcement over time. Additionally, it is possible that some staff documented certain items in the second cycle primarily in response to audit feedback, without fully understanding their clinical importance. Once the immediate audit pressure diminished, these items may have been deliberately deprioritized.

Comparable trends have been reported in other quality improvement studies, where initial enhancements in documentation quality diminished without robust, ongoing supervision and continuous quality assurance activities [7-9]. An alternative explanation for this decline may be related to staff perceptions of necessity and relevance. Some clinicians may have documented certain fields in the second cycle primarily in response to the initial audit feedback, without fully understanding their clinical importance. Once the immediate sense of oversight diminished, these fields were deprioritized in the third cycle. This highlights that sustainability challenges may stem not only from workload or fatigue but also from a lack of intrinsic motivation. Addressing this requires ongoing education that emphasizes the clinical rationale behind each documentation item, ensuring staff recognize its value for patient care and are motivated to sustain practice change. These findings reinforce the importance of quality improvement initiatives incorporating iterative monitoring, supportive supervision, and possibly simplification of documentation to maintain sustainability and reduce user burden.

Core identifiers such as patient name, age, weight, and admission date achieved near-complete documentation rates (48/50, 96.0% each in the final cycle), indicating successful embedding of these practices into routine care. Nevertheless, critical gaps persisted in the recording of vital signs, allergy status, and discharge status (all 0/50, 0.0% throughout cycles), mirroring persistent documentation challenges commonly observed in low- and middle-income country health systems. Future interventions should prioritize addressing these gaps, potentially through mandatory fields, structured checklists, or integration with electronic medical record systems to facilitate comprehensive and standardized data capture.

Stakeholder feedback was generally positive regarding the acceptability of the new admission sheet and the associated training program. However, users highlighted the ongoing need for continuous education, simplification of documentation fields, and additional support mechanisms to ensure consistent and sustained adherence. Integrating user-centered design principles and leveraging digital technology where feasible may facilitate improved compliance and long-term sustainability of documentation quality.

This study had several limitations. The single-center design may limit the generalizability of findings to other healthcare settings with different resources or patient populations. The audit assessment relied solely on the completeness of available documentation forms, which may not fully capture undocumented clinical activities. The relatively short duration between audit cycles may have been insufficient to fully assess the long-term sustainability of documentation improvements. Additionally, staff turnover and variable engagement potentially influenced the consistency of documentation practices. Finally, qualitative feedback was limited; more comprehensive exploration of barriers and facilitators to documentation adherence would strengthen understanding and inform future quality improvement efforts.

## Conclusions

The implementation of a standardized inpatient admission sheet in the Department of Pediatrics at Atbara Teaching Hospital, accompanied by targeted staff training and iterative audit-feedback cycles, led to significant improvements in the completeness and quality of patient admission documentation. Key demographic and presenting complaint data began to be captured more consistently, contributing to enhanced clinical communication and patient management. However, the observed regression in detailed clinical history and physical examination documentation during the later audit cycle highlights the challenges of sustaining comprehensive record-keeping without ongoing reinforcement.

## Figures and Tables

**Figure 1:**
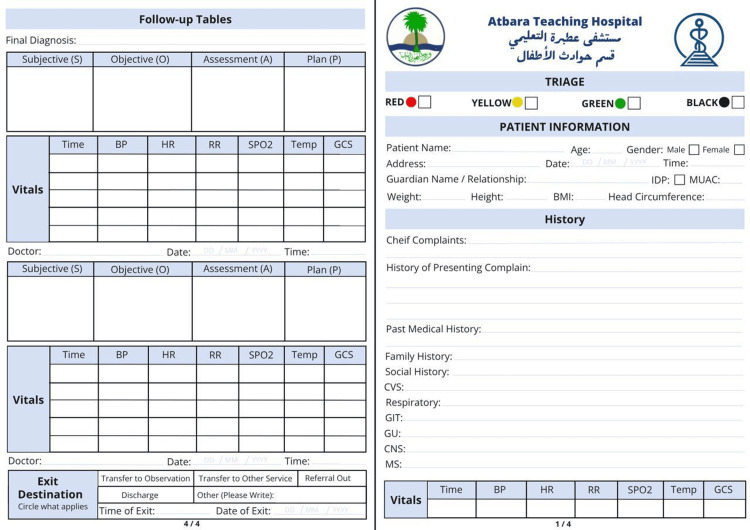
Front page of the new Inpatient Admission Sheet

**Figure 2:**
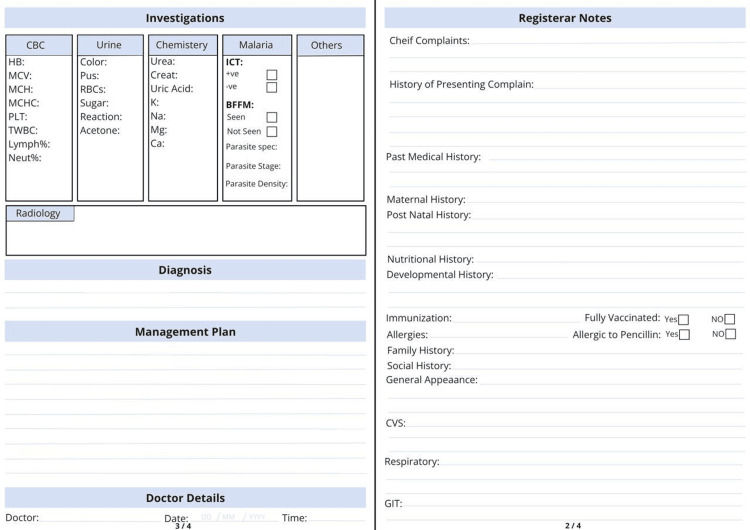
Back page of the new Inpatient Admission Sheet
